# Development of a deep learning based approach for multi-material decomposition in spectral CT: a proof of principle in silico study

**DOI:** 10.1038/s41598-025-09739-9

**Published:** 2025-08-06

**Authors:** Jayasai R. Rajagopal, Saikiran Rapaka, Faraz Farhadi, Ehsan Abadi, W. Paul Segars, Tristan Nowak, Puneet Sharma, William F. Pritchard, Ashkan Malayeri, Elizabeth C. Jones, Ehsan Samei, Pooyan Sahbaee

**Affiliations:** 1 Carl E. Ravin Advanced Imaging Laboratories and Center for Virtual Imaging Trials, Department of Radiology, Duke University Medical Center, Durham, NC 27705 USA; 2 Radiology and Imaging Sciences, National Institutes of Health Clinical Center, Bethesda, MD 20892 USA; 3 Siemens Healthineers, Princeton, NJ 08540 USA; 4 Siemens Healthineers AG, Siemensstr. 3, 91301 Forchheim, Germany; 5 Siemens Healthineers, 19335 Malvern, PA USA

**Keywords:** Material decomposition, Spectral CT, Computed tomography, Material decomposition, Simulation, Deep learning, Three-dimensional imaging, Tomography, Medical imaging

## Abstract

Conventional approaches to material decomposition in spectral CT face challenges related to precise algorithm calibration across imaged conditions and low signal quality caused by variable object size and reduced dose. In this proof-of-principle study, a deep learning approach to multi-material decomposition was developed to quantify iodine, gadolinium, and calcium in spectral CT. A dual-phase network architecture was trained using synthetic datasets containing computational models of cylindrical and virtual patient phantoms. Classification and quantification performance was evaluated across a range of patient size and dose parameters. The model was found to accurately classify (accuracy: cylinders – 98%, virtual patients – 97%) and quantify materials (mean absolute percentage difference: cylinders – 8–10%, virtual patients – 10–15%) in both datasets. Performance in virtual patient phantoms improved as the hybrid training dataset included a larger contingent of virtual patient phantoms (accuracy: 48% with 0 virtual patients to 97% with 8 virtual patients). For both datasets, the algorithm was able to maintain strong performance under challenging conditions of large patient size and reduced dose. This study shows the validity of a deep-learning based approach to multi-material decomposition trained with in-silico images that can overcome the limitations of conventional material decomposition approaches.

## Introduction

Spectral CT represents an extension of CT imaging capabilities^1^most recently through photon-counting CT (PCCT)^2,3^ with improved dose efficiency. A technique that exemplifies such increased capacity is material decomposition, which exploits the difference in signal responses of different materials across different x-ray energy levels to identify and quantify materials^4^. There have been multiple clinical applications of this technique including identifying different types of renal tumors^5^differentiating the composition of kidney stones^6,7^perfusion imaging^8,9^and calcium removal^10,11^.

Material decomposition is an active area of research to ensure robust implementation. Challenges include the need for calibration per acquisition condition to achieve precise diagnostic performance. Further, material decomposition is sensitive to noise and systemic errors in initial CT acquisition. These errors propagate into material maps and causes inaccuracy in reported values^12,13^. As the number of materials of interest increases, the information about specific material quantities becomes confined to increasingly smaller volumes in the vector space defined by initial images. An additional challenge is the issue of photon starvation. When the imaged object is large or contains some dense materials, the number of photons detected in certain regions of interest decreases which leads to an overall decrease in image quality and in material decomposition performance^14,15^. Most importantly, the performance of conventional material decomposition algorithms is dependent on patient size and applied dose level. While changes in acquisition protocol can minimize the impact of these challenges, clinical scans remain under-optimized for material differentiation tasks across different clinical scenarios.

Machine learning and artificial intelligence techniques^16,17^ can address some of the above challenges. Further, a recent FDA announcement^18^ has highlighted the importance of computational modeling and simulations in medical device submissions. That includes virtual clinical trial platforms^19^ that provide a pathway for in-silico evaluations of specific clinical scenarios with a known ground truth and conditions that would be difficult to replicate in practice, such as the introduction of contrast materials not yet approved by the FDA. The goal of this work is to determine the feasibility of a machine learning based approach to address the limitations of conventional material decomposition. In this proof-of-principle study, we developed an algorithm that uses machine learning techniques to improve the performance of material decomposition. A physics guided simulation platform that replicates scanner specific features was used to create image datasets of cylindrical and virtual patient phantoms containing materials of interest. A machine learning algorithm was then trained and validated on these models for clinically relevant material decomposition tasks across a variety of dose and size conditions.

## Methods

**Fig. 1:**
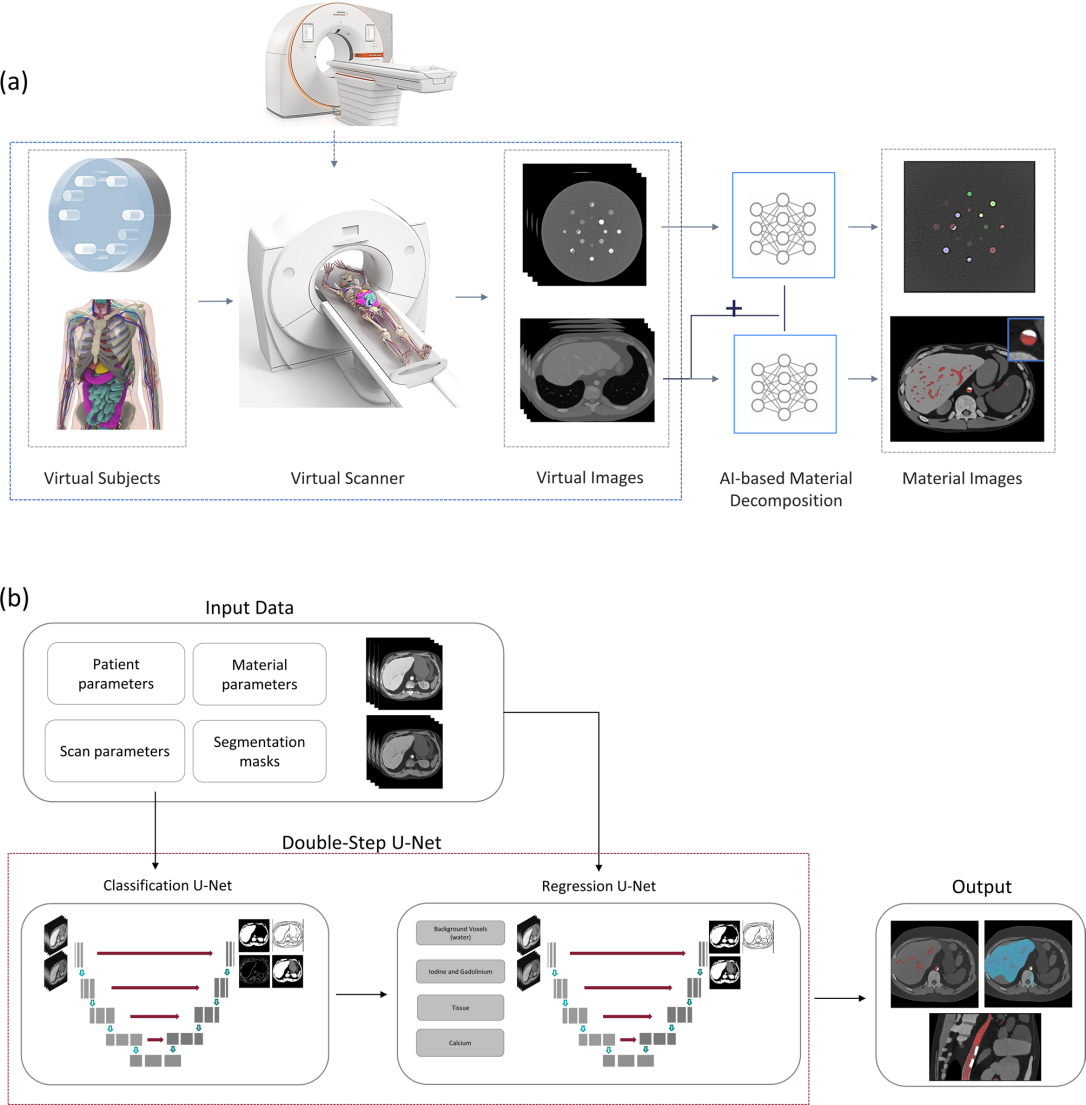
(**a**) Flowchart of the process used in this paper. The process initially begins with computational phantoms, either cylindrical or virtual patient. A CT simulation platform (DukeSim) is used to simulate image acquisition with a photon-counting CT system. Reconstructed images are then passed to a series of neural networks for material decomposition which results in the final material maps. (**b**) Network architecture used for the material decomposition model. First the feature network is trained to detect different material types, and then the material concentration model is trained to predict the concentrations of each material component.

The overall workflow for this study is shown in Fig. 1a. Computational phantoms, either cylindrical or virtual patient, were assigned material properties including target materials of interest (iodine, gadolinium, and calcium). A validated CT simulation platform was then used to simulate image acquisition for both datasets. Reconstructed images were fed into a series of deep learning models to perform material decomposition. The final output was a series of material maps. Section 2.1 outlines the theory of material decomposition. Section 2.2 and 2.3 provide further detail on the two datasets used in this study. Section 2.4 describes the CT simulation platform. Section 2.5 explains the deep learning model. Section 2.6 covers the evaluation and statistics.

This work did not involve any human subjects or animal subject research. Human Ethics and Consent to Participate: not applicable.

### Theory of material decomposition

Spectral computed tomography (CT) leverages the energy-dependent attenuation properties of X-ray photons to differentiate between materials based on their composition. By acquiring measurements at multiple energy spectra, spectral CT enables material decomposition, wherein the composition of scanned objects is estimated from the measured attenuation profiles. When a voxel contains a mixture of known materials, its total linear attenuation coefficient can be modeled as a weighted sum^20^:

$$\:{\mu\:}_{tot}=\:{\sum\:}_{i}{w}_{i}{\mu\:}_{i}$$

where µ_tot_ is the total attenuation coefficient, µ_i_ is the attenuation coefficient of the i-th basis material at a given energy, and w_i_ represents its fractional contribution. Spectral CT acquisitions produce multiple energy-resolved measurements for each voxel, denoted as a vector X ∈ ℝ^M^. These measurements are modeled as a linear combination of material basis coefficients Y ∈ ℝ^N^, using a sensitivity matrix Γ ∈ ℝ^M × N^:

$$X{\text{ }} = {\text{ }}\Gamma Y$$

The matrix Γ contains known energy-dependent attenuation values for each basis material and is typically calibrated using a phantom with predefined material concentrations. The number of materials that can be accurately estimated depends on the number of energy channels acquired and the structure of the sensitivity matrix.

In this study, we adopt a deep learning–based material decomposition framework. The approach consists of two stages: a classification step to identify the materials present in each voxel, followed by a regression step to estimate their respective concentrations. This formulation enables the model to capture nonlinear relationships in spectral data and offers improved flexibility over conventional decomposition techniques.

### Cylindrical phantom dataset

The first simulated dataset was a series of cylindrical phantoms with a water background containing multiple cylindrical inserts. Phantoms were generated at four sizes (20, 28, 36, and 42 cm diameter) chosen to represent the typical range of abdominal diameters that could be expected in a clinical population. Each phantom had two rings of eight inserts centered at a radius of 3.5 and 7 cm from the center. Inserts were grown to a random size between 1.5 and 3.0 cm around seed points to represent variable sizes of vasculature. Four of the sixteen inserts were randomly selected in each phantom to be split into two material classes. Three phantoms were generated at each size for a total of twelve unique cylindrical phantoms.

Inserts were filled with materials belonging to one of seven material classes: soft tissue, iodine (0-7.5 mg/mL), gadolinium (0–5 mg/mL), calcium (0-300 mg/mL), iodine/gadolinium mixture, iodine/soft tissue mixture (0-2.5 mg/mL) and gadolinium/soft tissue mixture (0–4.0 mg/mL). Material concentration ranges were chosen to represent similar levels of enhancement for iodine and gadolinium. Concentration for iodine/soft tissue mixtures were chosen to represent peak enhancement in the delayed phase of the liver^21^. All concentrations were randomly assigned to specified inserts across all phantom sizes.

Dose levels were adjusted for each phantom size based on a pilot study to characterize noise as a function of size and tube current. A series of water phantoms at five sizes (20, 25, 30, 35, 40 cm diameter) were simulated at five tube current levels (25, 50, 100, 150, 200 mAs). A region-of-interest (ROI) was drawn at the center of each image and the standard deviation of pixel values taken as a noise measure. A linear fit of noise relative to phantom size and tube current was calculated for both low and high energy images. Noise levels that were matched across different phantom sizes were referred to as isonoise levels. A detailed description of the parameters can be found in Table 1.

**Table 1:** Phantom size, dose, and associated noise parameters for cylindrical and virtual Phantom simulations. Noise levels estimated from linear fitting procedure.

Group	Variable	Cylindrical phantom	XCATs
Small	Phantom diameter (cm)	20	23.0–25.7
	Tube currents (mAs)	44; 110; 150	167–221; 205–258
	Dose (CTDIvol, mGy)	3.4; 8.6; 11.7	13.1–17.2; 16.0–20.2
	Isonoise fit (HU)	8.5; 5.0; 3.0	5.0; 3.0
Medium	Phantom diameter (cm)	28	27.5–32.6
	Tube currents (mAs)	46; 113; 198	103–202; 188–287
	Dose (CTDIvol, mGy)	3.6; 8.8; 15.4	8.0–15.8; 14.7–22.4
	Noise fit (HU)	16.5; 13.0; 8.5	13.0; 8,5
Large	Phantom diameter (cm)	36	33.3–36.9
	Tube currents (mAs)	106; 153; 200	100–171; 148–218
	Dose (CTDIvol, mGy)	8.3; 11.9; 15.6	7.8–13.3; 11.5–17.0
	Isonoise fit (HU)	21.5; 19.0; 16.5	19.0; 16.5
Extra large	Phantom diameter (cm)	42	38.0–43.3
	Tube currents (mAs)	145; 184; 221	107–210; 145–248
	Dose (CTDIvol, mGy)	11.3; 14.4; 17.2	8.4–16.4; 11.3–19.3
	Isonoise fit (HU)	25.5; 23.5; 21.5	23.5; 21.5

### In-silico patient dataset

The second simulated dataset consisted of a series of phantoms from the XCAT database^22^. Thirty adult abdominal models were chosen to represent a realistic clinical population. Patients were divided into four size groups (small, medium, large, extra large) based on abdominal diameter and matched to the size groups from the cylindrical phantom dataset. Each phantom had two calcified plaques inserted into the aorta at the level of the liver. A dual-contrast scenario was simulated with an initial bolus of gadolinium followed by a delayed injection of iodine. Sufficient time was assumed for gadolinium (0.5–3.5 mg/mL) to be primarily visible in the renal collecting systems while iodine was present in vasculature (4–7 mg/mL aorta, 2–5 mg/mL intra-organ vasculature). Two dose levels were calculated using the noise linear fit for each patient representing full and reduced dose acquisitions. A detailed description of the parameters can be found in Table 1.

### CT simulator and scanner setup

Image simulation was performed using DukeSim (Duke University, USA)^23^. The simulator enables rapid simulation of CT images using computational phantoms and is able to replicate scanner-specific geometry, physics, and protocol settings^24,25^ and has been validated for the simulation of PCCT systems^26,27^. A clinical PCCT scanner^18,28^ (NAEOTOM Alpha, Siemens Healthineers, Forchheim, Germany) was simulated in this study. The scanner was simulated using Quantum Plus (Qplus) mode which uses a 144 × 0.4 mm collimation and 0.4 × 0.4 mm in-plane pixels.

Imaging protocols were adapted from the manufacturer’s recommended settings for abdominal-pelvic imaging. All simulations were completed with a tube voltage of 120 kV, a rotation time of 0.5 s, a pitch of 0.8, energy thresholds of 20 and 65 keV and fixed tube current. The lower energy threshold functions as a noise floor to suppress electronic noise while the upper threshold separates the signal into low and high energy photon bins. Projections were reconstructed using an offline reconstruction program (ReconCT v. 15.0.55067.0) with a 30 cm field-of-view, 2.0 mm slice thickness, 512 × 512 matrix size, and a quantitative soft tissue kernel (Qr40f).

### Deep learning architecture and structure

The deep learning model was trained in two phases (Fig. 1b). In the first phase, a feature detection network was trained using a U-Net architecture^29^. In the second phase, a separate U-Net network was trained, which took the feature maps from the first network as inputs and returned four outputs corresponding to the concentrations of iodine, gadolinium, calcium and a probability map for the presence of tissue. These models are described below.

#### Feature network

The feature network took four different image patch channels as input: two channels corresponding to the low and high energy CT images, one channel containing the distance of each voxel from the center of the phantom, and another channel containing the radial distance of each voxel from the edge of the phantom. The two distance maps together encoded information about the relative position of the voxel in reference to phantom size. The image patches were taken to be of a fixed size of 64 × 64 voxels, and the entire image was decomposed into overlapping patches with an overlap of 16 pixels in each direction. The output of the network was the probability that each voxel belongs to one of 4 different classes: (i) background voxels (water), (ii) presence of calcium, (iii) presence of any mixtures of iodine and gadolinium, without tissue, and (iv) voxels containing tissue. During training, only the patches containing the above materials were treated as positive samples. In the virtual patient datasets, only vascular calcium (inside aorta) was treated as positive for calcium. All other materials containing calcium, such as bones or the spine, were all treated as background material for training the network.

The U-Net architecture was made of a convolutional architecture with convolutions of kernel size 3 × 3 pixels combined with a batch normalization layer and a rectified linear (ReLU) activation model. In the encoder path, the spatial resolution is progressively reduced by a factor of 2 at each level while the number of feature channels is increased, starting with 16 channels in the first step. Specifically, the feature map dimensions evolve as follows:

$${\text{16x}}\left( {{\text{64x64}}} \right) \to {\text{32x}}\left( {{\text{32x32}}} \right) \to {\text{64x}}\left( {{\text{16x16}}} \right) \to {\text{128x}}\left( {{\text{8x8}}} \right) \to {\text{256x}}\left( {{\text{4x4}}} \right)$$

In the decoder path, the model uses transposed convolutional layers to increase the image resolution from 4 × 4 voxels to 16 channels of 64 × 64 voxels. In the final layer, the 16 channels were reduced to 4 channels using a convolutional operator with a softmax layer to estimate the probabilities of each material type. This model is trained until the validation loss (cross-entropy for predicting material classes) does not decrease any further.

### Material concentration model

The second network took as an input the 16 channels of output preceding the final convolutional layer from the feature network, and returned four channels of output: (i) concentration of iodine, (ii) concentration of gadolinium, (iii) concentration of calcium without tissue, and (iv) probability of the presence of tissue. During the training of the material concentration model, the network weights of the first U-Net model (described above) were frozen, and only the parameters of the material concentration model were updated.

The material concentration model also used a U-Net architecture similar to the feature network, with similar parameters in terms of the patch sizes and convolutional kernel shapes. The model was trained using an input patch size of 64 × 64 voxels, with a learning rate of 1.0e-4, a batch size of 256, using the Adam optimizer, and learning rate was controlled by reducing LR on plateau by 0.1.

#### Model training and cross-validation

The model was trained using twelve-fold cross-validation. For each fold, three phantoms were randomly held out of the dataset and the remaining thirty-three used for model training and validation. Four phantoms were used to evaluate validation loss and the remaining twenty-nine used for training. Each fold was run for 100 epochs with an early control for diminishing validation loss.

### Evaluation and statistical considerations

For the cylindrical phantom dataset, analysis was focused on the relationship between material quantification of iodine, gadolinium and calcium in material maps when compared to dose and size parameters. Ground truth masks were used to segment regions of interest (ROI) containing either iodine, gadolinium, or a mixture, and the mean and standard deviation of the voxels within the ROI were calculated. Performance was evaluated between ground truth and estimated values with correlation, root mean squared error (RMSE), and mean absolute percentage difference (MAPD) as figures of merit. Mean absolute percentage error was calculated for those cases where the ground truth concentration was greater than zero.

For the virtual patient phantom dataset, analysis was focused on multilabel classification performance and regression performance of the iodine/gadolinium pair. The multilabel classification accuracy, sensitivity, and specificity of the model were assessed across all materials. Regression results were evaluated similarly to the cylindrical phantom dataset between ground truth and estimated values. Figures of merit included correlation, RMSE, and MAPD.

Prediction dependence on size and projected noise was characterized by considering mean absolute difference for each value of each variable. Further, linear mixed effects models were fit to the predicted concentrations for each material with phantom size and tube current as fixed effects and possible correlation between phantom size and tube current. An analysis of variance was applied to the residuals of the linear mixed effects models to evaluate variability due to tube current and phantom size.

## Results

### Cylindrical phantom results

**Fig. 2:**
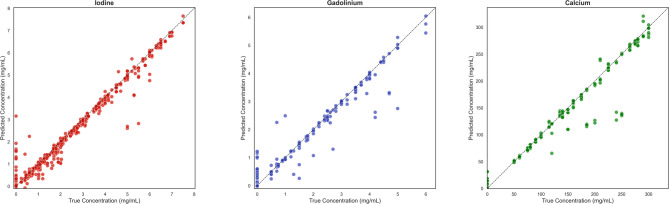
True concentration (x-axis) vs. predicted concentration (y-axis) separated by material (iodine – left, gadolinium – middle, calcium – right). Each data point represents the concentration within a region of interest averaged over fifteen slices. Values systematically fell below the true = predicted line due to underestimation in mixtures and overlap in adjacent iodine/calcium regions.

Across all acquisition conditions, the model provided sensible performance for iodine, gadolinium, and calcium quantification (Fig. 2). In terms of correlation, predicted and ground truth concentration measures were closely matched for iodine (98.3%), gadolinium (98.0%), and calcium (98.6%). Iodine and gadolinium had low RMSE values of 0.36 mg/mL and 0.24 mg/mL respectively. Calcium had a larger RMSE of 13.34 mg/mL but was lower in magnitude compared to the range of non-zero values for calcium. In terms of MAPD, the model performed well for all three materials with values of 10.8% for iodine, 10.9% for gadolinium, and 7.6% for calcium. Prediction bias due to material concentration was slightly negative for all three materials within a range of 0.8 mg/mL for iodine, 1.2 mg/mL for gadolinium and 75 mg/mL for calcium. We attribute this to the relatively small sample of the training dataset, and the imbalance between the number of positive (containing materials of interest) and negative regions (background) in the images.

### In-silico patient results

**Fig. 3:**
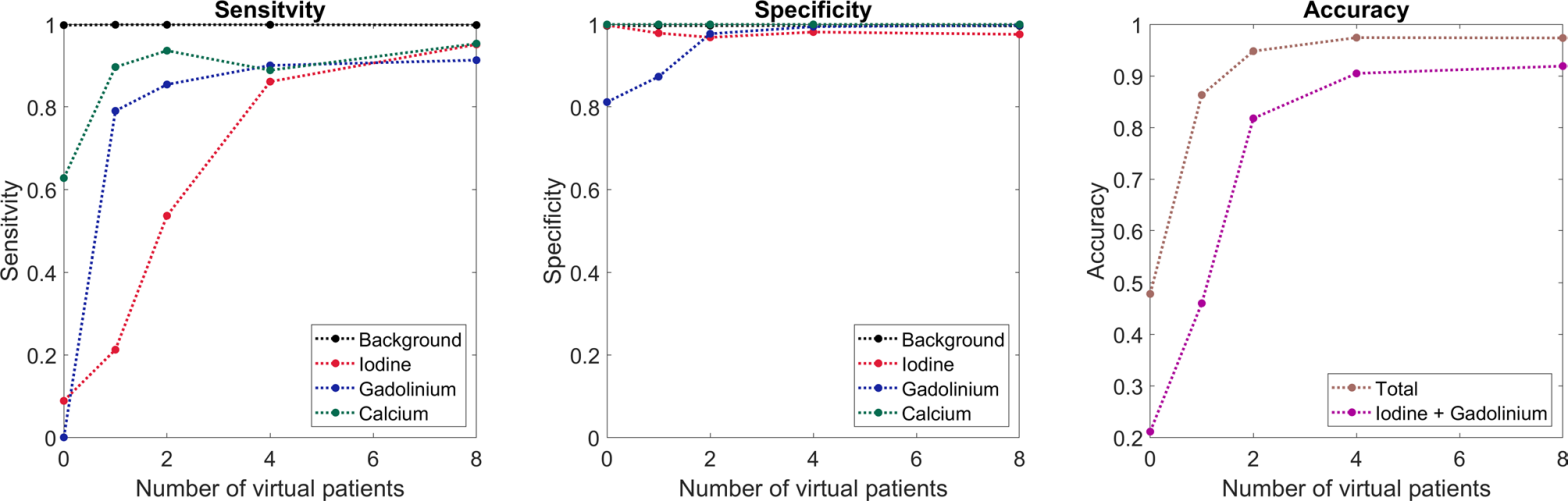
Sensitivity (left), specificity (middle), and accuracy (right) of classification model performance based on the number of virtual patient phantoms included in training data (x-axis). Colors indicate class label (background tissue – black, iodine – red, gadolinium – blue, calcium – green) for sensitivity and specificity plots. For accuracy plot, total (brown) and iodine and gadolinium (purple) accuracy was tracked. Inclusion of anthropomorphic models significantly increased performance for iodine and gadolinium classification.

Overall, classification model performance was good for background, iodine, gadolinium, and calcium voxels and improved as additional virtual patient models were included within the training data (Fig. 3). Accuracy across all material classes improved from 47.8% with no virtual patients to 97.3% with 8 virtual patients in the training dataset. For the iodine/gadolinium pair, accuracy improved from 21.1% with no virtual patients to 91.9% with 8 virtual patients.

**Fig. 4:**
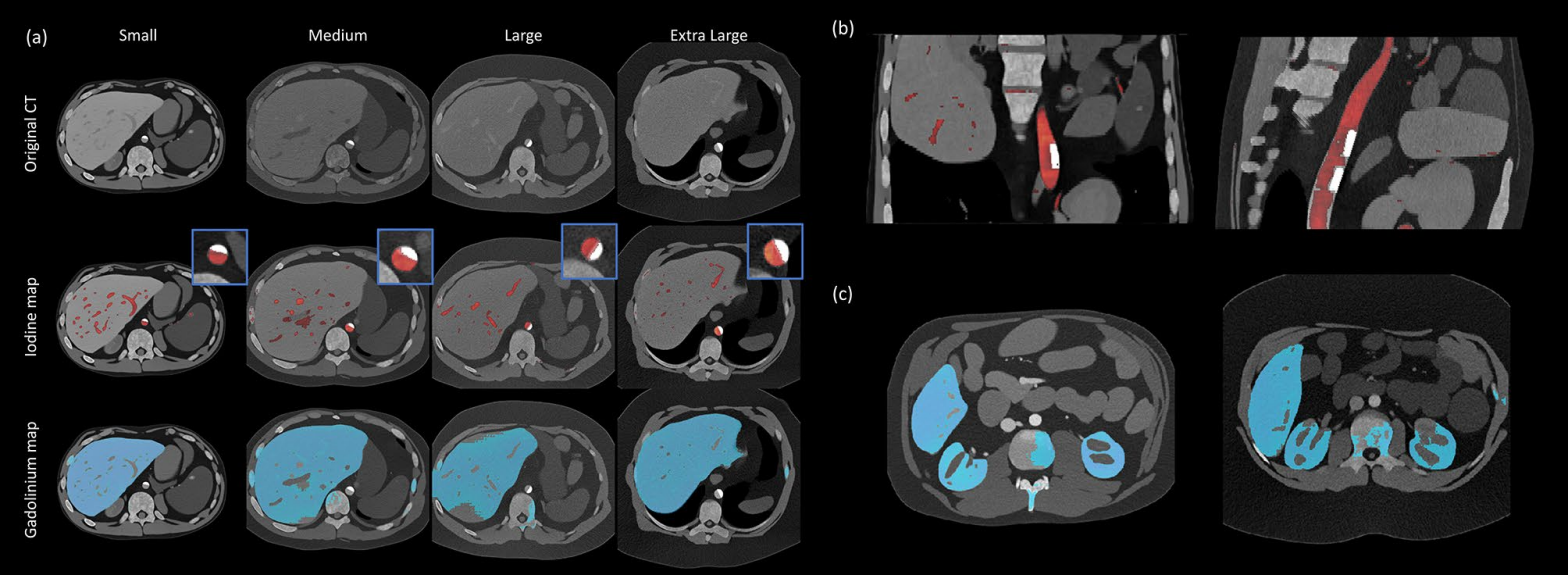
(**a**) Visualization of quantification outputs from hybrid model. Virtual patients organized by size group (columns). First row shows low threshold CT image (window width/level – 300/500). Second row shows iodine map and third row shows gadolinium map. Zoomed in sections in the second row show calcified aortic plaque and iodine in lumen. Cases show conditions that were challenging for model performance including bone misclassified as contrast material and contrast enhanced tissue classified as background. (**b**) A coronal view of virtual patient aortas showing calcified plaque (white regions) and iodine in lumen (red). Image shown of virtual patient from the small (left) and medium (right) size groups.(**c**) Visualization of virtual patient liver and kidneys with gadolinium (blue). Images shown of virtual patients from the small (left) and extra-large (right) size groups. Both cases show challenging conditions for the algorithm where parts of the spine were classified as contrast material.

**Fig. 5:**
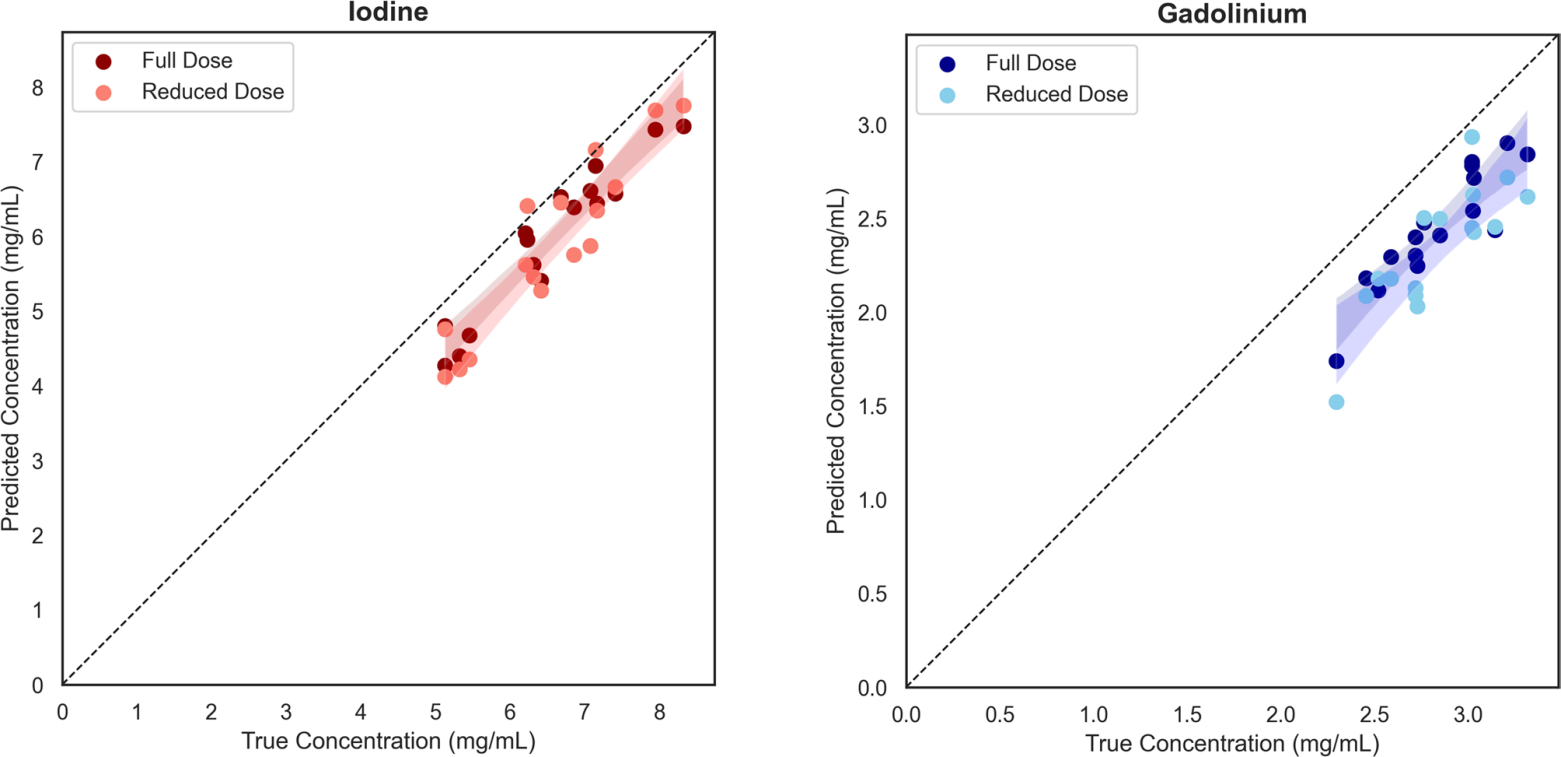
True concentration (x-axis) vs. predicted concentration (y-axis) for the virtual patient dataset separated by material (iodine – left, gadolinium – right). Shade represents different dose levels (darker – full dose, lighter – reduced dose). Each data point represents the concentration within a single phantom/dose combination. The shaded regions represent the 95% confidence intervals of the material concentrations.

Visual examples of the final quantification outputs can be found in Fig. 4. For quantification, model performance was variable (Fig. 5). In terms of correlation, predicted and ground truth measures were closely related for iodine (0.940) but less strongly correlated for gadolinium (0.487). RMSE was higher compared to values in the cylindrical phantom dataset for both iodine (0.73 mg/mL) and gadolinium (0.50 mg/mL). In terms of MAPD, performance was comparable to the cylindrical phantom data for both iodine (10.1%) and gadolinium (14.5%). Prediction bias due to material concentration was negative for both materials and within a range of 1.5 mg/mL for iodine and 1.2 mg/mL for gadolinium.

### Overall performance results

**Fig. 6:**
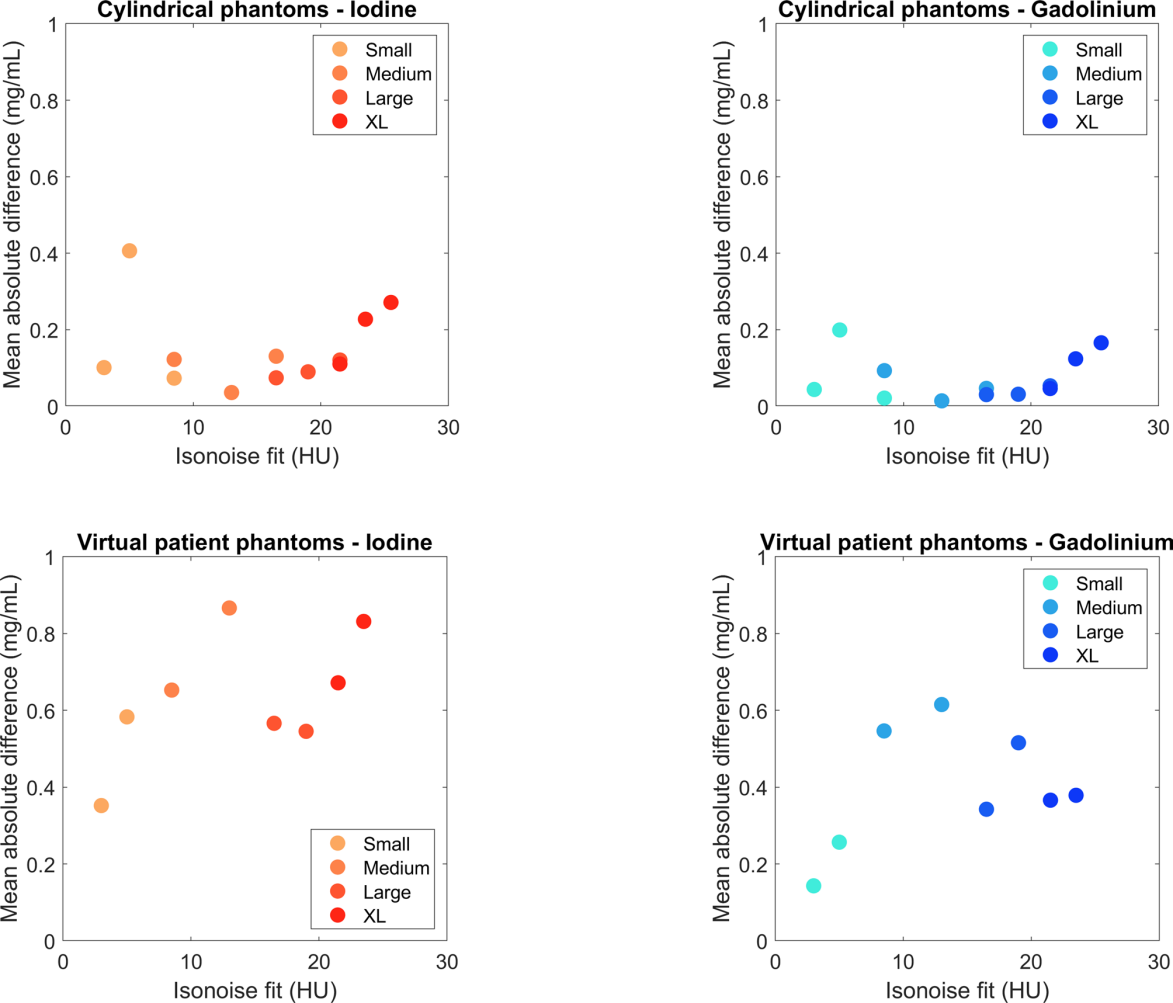
Mean absolute difference (y-axis) of grouped datasets for both cylindrical (top row) and virtual patient (bottom row) phantoms. Values are plotted against the isonoise fit value (x-axis) used to standardize exposure across phantom sizes. Each shade represents a different size of phantom.

When comparing performance relative to phantom size and dose conditions, both datasets showed strong overall performance (Fig. 6). Overall mean absolute difference was below 1 mg/mL for all datasets. The virtual patient dataset showed higher mean absolute difference than the cylindrical data. Both datasets showed a slight increase in mean absolute difference as dose was decreased (0.12–0.16 mg/mL for cylinders and 0.52–0.54 mg/mL for virtual patients). For the anthropomorphic phantom dataset only, there was a slight dependence on phantom size as well.

The linear mixed effects model and analysis of variance results for both datasets are summarized for each material in Table 2. For the cylindrical phantom dataset, phantom size and tube current had a limited impact (< 0.02 mg/mL per cm and mAs for iodine and gadolinium; <1.0 mg/mL per cm and mAs for calcium) on model predictions. Tube current did not have an impact on predictions across materials while phantom size had a high p-value for iodine and p-values near 0.05 for both gadolinium and calcium. For the virtual patient dataset, phantom size and tube current had a limited impact (< 0.02 mg/mL per cm and mAs for iodine and gadolinium) on model predictions. Tube current did not have an impact on predictions for either material while phantom size had a high p-value for both iodine and gadolinium.

**Table 2:** Summary of linear mixed effects models and analysis of variance of cylindrical phantoms and virtual patient phantoms for each material prediction. Phantom size and tube current treated as fixed effects. Analysis of variance was evaluated for the residuals of the linear mixed effects model.

Cylindrical phantoms		Iodine	Gadolinium	Calcium
Intercept	Estimate	1.05	0.25	57.45
	SE	0.47	0.11	13.98
	Lower	0.12	0.02	30.04
	Upper	1.98	0.47	84.86
	F-stat	4.91	4.72	16.88
	P-value	0.03	0.03	0.00
Phantom Size	Estimate	0.02	0.01	-0.92
	SE	0.01	0.00	0.46
	Lower	-0.01	0.00	-1.82
	Upper	-0.04	0.01	-0.01
	F-stat	1.62	3.48	3.97
	P-value	0.20	0.06	0.05
Tube Current	Estimate	0.00	0.00	0.02
	SE	0.00	0.00	0.03
	Lower	0.00	0.00	-0.04
	Upper	0.00	0.00	0.09
	F-stat	0.01	0.76	0.51
	P-value	0.92	0.38	0.48

Virtual patient phantoms		Iodine	Gadolinium
Intercept	Estimate	5.94	2.61
	SE	1.04	0.26
	Lower	3.81	2.07
	Upper	8.06	3.14
	F-stat	32.63	99.1
	P-value	0.00	0.00
Phantom size	Estimate	0.02	0.01
	SE	0.03	0.01
	Lower	-0.04	-0.01
	Upper	0.08	0.02
	F-stat	0.35	0.54
	P-value	0.56	0.47
Tube current	Estimate	0.00	0.00
	SE	0.00	0.00
	Lower	0.00	0.00
	Upper	0.00	0.00
	F-stat	0.00	0.00
	P-value	0.99	0.99

## Discussion

Spectral CT has expanded the capabilities of computed tomography by acquiring images at multiple energy levels. This enables material decomposition where materials are identified and quantified based on their unique signal responses at different energies. Accurate material decomposition is important for clinical tasks including tumor characterization, perfusion imaging, and calcium removal. While successful for many clinical tasks, conventional techniques face significant challenges. In this study, a deep learning-based approach is used to provide a more adaptive and reliable solution to address traditional material decomposition’s major challenges.

Conventional material decomposition methods rely on an intricate process of spectral calibration as there cannot be a perfect solution to the series of linear differential equations that project CT images into a material quantity domain^30,31^. Even minor systematic, stochastic, or human errors can be amplified by this process, leading to significant inaccuracies in the final material maps. Calibrating across varying energy spectra, photon flux, and spatial positions is challenging and can be further complicated by issues like charge sharing and pulse pile-up in photon-counting CT^32^. These complexities make spectral calibration time-consuming and prone to variability across different imaging conditions and setups which can undermine reliability or limit the clinical applicability of the method. Complexity is further increased when extending base materials to greater than two, as the number of energy thresholds needs to be increased, for example four thresholds for four materials as in this study. Finally, materials and thresholds must be carefully chosen for optimal signal extraction^33,34^. This comes at the cost of degrading signal quality in each acquired threshold image leading to poor quantification results.

Conventional methods use voxel-level data making them highly dependent on the quality of acquired CT images. High image noise and photon starvation, which can be caused by clinical dose limitations and larger patient sizes, undermine the reliability of material quantification. Earlier studies have described the impact of patient size and dose on material characterization. Pelgrim et al.^14^ imaged tubes of iodine with different sized phantoms with two dual-energy CT scanners and found that larger phantom sizes increased variability in iodine measurements. Euler et al.^35^ scanned lesion models of iodine at different size and dose levels across a range of dual-energy CT scanners and reported that increasing size and decreasing dose both had a significant impact on error. Finally, Lambert et al.^15^ imaged different materials, including iodine, gadolinium, and calcium, in a phantom with variable size fat rings. They found that while CT numbers decreased with phantom size, the dual-energy ratios between most material pairs did not significantly change suggesting that material separation should still be possible at larger sizes depending on material pairs. While the spectral CT technology used in our work differed from those in earlier works, the same challenges of large patient size and reduced dose would challenge a conventional material decomposition approach.

Our proposed deep learning method addresses the limitations of conventional approaches in several ways. First, it eliminates the need for a base material matrix or spectral calibration by bypassing the assumption of linear dependencies of differential equations and attenuation coefficients. Second, it incorporates contextual information beyond individual voxels, such as relative voxel positions, acquisition parameters, and local neighborhood statistics, which helps mitigate reduced signal quality due to large object sizes or low dose levels. By leveraging prior anatomical information and learned patterns from training data, the model enhances material identification and quantification, allowing for effective multi-material decomposition using only two energy levels. Careful selection and curation of datasets further optimize the model’s performance for specific clinical tasks, enhancing its clinical utility.

Deep learning algorithms are known to benefit strongly from increasing dataset sizes available for training^36^. However, obtaining large real-world datasets with known material properties, including material concentrations, is very difficult. In-silico approaches, such as the one presented in our manuscript, provide a way to generate physics-based ground truth spanning a broad range of input conditions expected in the real world. This enables strong models to be first built using datasets with solid ground truth, followed by fine-tuning on smaller real-world datasets if needed.

We used dual U-net architecture to provide a stable backbone for our task. Material decomposition was separated into two tasks, classification and quantification, requiring a dual-network architecture. The first network identified voxels containing materials of interest and excluded background voxels. The second network quantified materials in identified voxels containing target materials. This approach improved quantification performance by improving data quality. Initially, models were trained with the cylindrical dataset to evaluate the feasibility of the approach. The training dataset was then extended to include virtual patients to account for the complex geometry of human anatomy. For both stages, datasets were designed to address the clinical utility of each material decomposition task. This approach based on hybrid dataset showed that a model trained on simpler geometry can be augmented to perform well on more complex geometry.

The overall model provided robust performance across both classification and quantification tasks. That was so not only under favorable conditions of a small phantom size and low noise, but also under more challenging conditions of a larger phantom size and higher noise. Nonetheless, there were difficulties under specific conditions. The voxels with a material combination that the model had not seen before, such as cortical bone in the spine and ribs, were misclassified as containing contrast agents. Additionally, voxels on the boundary of organs were sometimes misclassified as not containing any contrast. This is expected as any model is constrained by the dataset that is used to train it.

The general goal of our study was to offer a proof-of-principle demonstrating the feasibility of a deep learning-based approach for basis material decomposition. While proven effective and applicable, this strategy requires additional refinements for clinical implementation. In particular, the use of experimental data will be important to assess real-world performance and generalizability as several practical factors can lead to inconsistencies between simulated training data and experimental test conditions. These include variations in phantom geometry, material composition and concentration, acquisition protocols, and reconstruction methods. Anticipating these challenges is essential when designing experimental studies and preparing data for model evaluation. A controlled and well-aligned experimental framework, together with broader and more diverse training data, both in vitro and clinical, will extend our findings towards a robust and generalizable material decomposition model.

An additional limitation of our study is the use of only two energy thresholds. While we anticipate that additional training data with more energy thresholds could improve accuracy and enable decomposition of voxels containing more than two mixed materials, our goal was to demonstrate that the number of materials decomposed is not linearly dependent on the number of energy thresholds. Further, the focus of this study was to develop an algorithm that was adaptive to variable size and dose conditions; other potential sources of variability were not explored. The general technique can be expanded to other factors including different acquisition and reconstruction conditions.

## Conclusion

This proof-of-concept study presents a deep learning-based approach that addresses the limitations of conventional material decomposition in spectral CT imaging. By bypassing spectral calibration and integrating contextual information, our model enhances material identification and quantification across various dose and size conditions. The dual-network architecture allows effective classification and quantification, even in challenging scenarios such as low dose levels and large patient sizes. These findings highlight the potential of deep learning to improve material decomposition accuracy and flexibility, paving the way for future clinical validation and broader application in spectral CT imaging.

## Data Availability

The data used to support the findings of this study are available from the corresponding author upon request.
